# Volunteering and health benefits in general adults: cumulative effects and forms

**DOI:** 10.1186/s12889-017-4561-8

**Published:** 2017-07-11

**Authors:** Jerf W. K. Yeung, Zhuoni Zhang, Tae Yeun Kim

**Affiliations:** Department of Applied Social Sciences, City University of Hong Kong, Kowloon, Hong Kong

**Keywords:** Other-oriented volunteering, Self-oriented volunteering, Cumulative effects, Health outcomes

## Abstract

**Background:**

Although the health benefits of volunteering have been well documented, no research has examined its cumulative effects according to other-oriented and self-oriented volunteering on multiple health outcomes in the general adult public. This study examined other-oriented and self-oriented volunteering in cumulative contribution to health outcomes (mental and physical health, life satisfaction, social well-being and depression).

**Methods:**

Data were drawn from the Survey of Texas Adults 2004, which contains a statewide population-based sample of adults (*n* = 1504). Multivariate linear regression and Wald test of parameters equivalence constraint were used to test the relationships.

**Results:**

Both forms of volunteering were significantly related to better health outcomes (odds ratios = 3.66% to 11.11%), except the effect of self-oriented volunteering on depression. Other-oriented volunteering was found to have better health benefits than did self-volunteering.

**Conclusion:**

Volunteering should be promoted by public health, education and policy practitioners as a kind of healthy lifestyle, especially for the social subgroups of elders, ethnic minorities, those with little education, single people, and unemployed people, who generally have poorer health and less participation in volunteering.

## Background

The beneficial effects of volunteering on health outcomes have been well documented. Research has found that participation in voluntary services is significantly predictive of better mental and physical health [1, 2], life satisfaction [3], self-esteem [3, 4], happiness [5, 6], lower depressive symptoms [4, 7], psychological distress [3, 8], and mortality and functional inability [8, 9]. As proved recently, the health benefits of volunteering are not due to self-selection bias. Recent longitudinal research did not support reverse causation, in which volunteering was significantly related to better health prospectively, and the reverse was not true [2].

What remains uncharted for the relationship between volunteering and health outcomes is pertinent to whether volunteering may have cumulative effects on health and what form of volunteering is preferable for promoting health benefits for volunteers [1, 7, 9]. Regarding the volunteering and health connection, the role accumulation perspective supports the position that a volunteer who concomitantly participates in different types of voluntary services can benefit his or her health most [2, 4, 5, 8]. The rationale is that role-related social privileges, resources, supportive network, coping skills, life meaning and gratitude accumulated through assuming multiple prosocial roles can be directly conducive to various health outcomes. In contrast, the scarcity thesis argues that simultaneous occupation of multiple roles would result in conflict and strain, which would compromise health [2, 4]. Therefore, it is worth investigating whether participation in multiple voluntary services contemporaneously, that is, the cumulative effects, would contribute to better health outcomes in the general public according to the relationship between volunteering and health.

This study is drawn from the meaning-fulfilling perspective that volunteering in general, by its prosocial and meaning-making nature, would positively contribute to health benefits [2, 10, 11]. However, different forms of volunteering are believed to have differential effects on health benefits [10–12]. Recent philanthropic research reports that the form of other-oriented volunteering has better health effects than does self-oriented volunteering in elderly people or in convenience samples [10, 11]. This is congruous with the meaning-fulfilling perspective with regard to the subtly different meaning-making processes that the two forms of volunteering engender. Other-oriented volunteering refers to helping others in need mainly by altruistic responsibilities and humanitarian concerns [2, 11]. These concerns can more effectively help accrue genuine supportive relationships and social integration, self-worth, a sense of mattering, and life meaning and therefore better contribute to health benefits [2, 11]. Self-oriented volunteering means the stress of volunteers on the reciprocity of volunteering, or volunteering affordance expressed by some scholars, that is, to seek benefits and enhance the volunteers themselves in return. Examples include strengthened social network and ties, understanding of self, acquisition of new skills, and career development [10–12]. In fact, such self-enhancing volunteerism is less effective in accruing meaningful and health-promoting benefits, e.g. supportive relationships and a sense of mattering, in the process of volunteering compared to other-oriented volunteering [2, 3, 11]. Thus, it is worth investigating whether the forms of other-oriented and self-oriented volunteering have different cumulative effects on health outcomes.

The current study aimed to investigate the cumulative effects of other-oriented and self-oriented volunteering, formed by participation in multiple pertinent voluntary services contemporaneously, on the health outcomes of perceived mental and physical health, life satisfaction, depression, and social well-being in a population-based sample of general adults. In addition, this study compared whether other-oriented volunteering has stronger health effects than does self-oriented volunteering.

## Methods

### Data and sample

The data used in this study were based on the Survey of Texas Adults 2004, which contains a statewide representative sample of 1504 community-dwelling adults aged 18 and over in Texas [13]. The sampling procedure was based on a modified random digit dialing design. A household-level cooperation rate of 37% and a respondent-level cooperation rate of 89% were obtained in the data-collection process. The survey was mainly conducted in English. Translation of survey instruments into Spanish and administration by a Spanish-speaking interviewer were applied when needed. Each computer-assisted telephone interview lasted about 30–35 min. Due to the overrepresentation of women, older adults, non-Hispanic Whites, and highly educated respondents in the original sample, the data were weighted to match the characteristics of the sample to the 2000 Texas population census estimates. The Survey of Texas Adults 2004 provided fruitful information on people’s participation in various types of voluntary services and health outcomes. Detailed socio-demographic data available in the survey can help adjust for confounding from the relevant background characteristics. The socio-demographic variables included in this study are gender, age, race/ethnicity, education, citizenship, marital status, number of children at home, employment status and family income. These background characteristics have been found influential on both volunteering and health outcomes in past research [1–3, 5, 9, 14].

### Measures

All measures employed for analysis in this study were self-reported by the adult participants. The information about these measures was mainly drawn from the sections of volunteering, physical health, mental health and demographic characteristics in the survey.

### Health outcomes

#### Mental health

In the Survey of Texas Adults 2004, a question was used to measure the mental health of the adult participants: “Overall, how would you rate your mental health at the present time? Would you say it is excellent, very good, good, fair, or poor?” The ratings are based on a 5-point scale (1 = excellent, 2 = very good, 3 = good, 4 = fair, and 5 = poor). For easy interpretation, the scale was reversely coded, meaning that higher scores represent better mental health.

#### Physical health

The adult participants in the survey were asked to respond to the question: “How would you rate your physical health at the present time? Would you say it is excellent, very good, good, fair, or poor?” The answers were used as a general indicator of physical health. The responses are also based on 5-point scale from 1 = excellent to 5 = poor. Again for better interpretation, the scale was reversely coded to indicate that higher scores represent better physical health.

#### Life satisfaction

The survey question used to rate participants’ levels of life satisfaction was: “How satisfied you are with your life overall?” The question was rated on a 4-point scale (1 = very satisfied, 2 = somewhat satisfied, 3 = not too satisfied, and 4 = not at all satisfied). The scale was reversely recoded, meaning that higher scores indicate better life satisfaction.

#### Depression

Five question items in the survey were used to measure participants’ depression in the past 30 days: “feeling sad”, “feeling hopeless”, “feeling everything was an effort”, “feeling worthless” and “had trouble breathing”. These items have been used to indicate depressive symptoms in prior research [7, 15]. They were rated on a 5-point scale from never (1) to several times a day (5), and a composite score was gathered by summing up the items. Cronbach’s α was .739 in this study.

#### Social well-being

Two question items in the survey were used to measure social well-being: “I am lacking companionship” and “I feel isolated from others”. These items are indicative of social integration and social acceptance and satisfaction about relationships with others [16, 17], which has been found influential on health [16]. The items were rated from strongly agree (1) to strongly disagree (5). The items were summed up to form a composite score. Higher scores imply better social well-being. Cronbach’s α was .717.

### Volunteering

#### Other-oriented volunteering

Recent philanthropic research indicates that volunteers simultaneously engage in various types of voluntary services; therefore, simply dichotomizing participants into volunteers and non-volunteers is inadequate [7, 14]. In this study, volunteering is classified into the two broad forms of other-oriented and self-oriented volunteering. Prior research indicates that volunteers of other-oriented motivation were more likely to volunteer in health, social, religious and other philanthropic services [11]. Hence, other-oriented volunteering was measured by summing up participation in the past 12 months in the voluntary services of health, education, religious groups, human services, public/social benefits, and youth development. In fact, these voluntary services explicitly bear other-regarding and altruistic features that, by their nature, show concern and care for the needs of others [2, 11]. The scores of this volunteering form range from 0 to 6, higher scores indicative of more participation in other-oriented volunteering.

#### Self-oriented volunteering

In this study, participation in the voluntary services of recreation, arts or culture, environment or animal welfare, work-related service, political campaign or movement, and other service simultaneously in the past 12 months was summed up to form self-oriented volunteering, the types of voluntary services bearing features of self-actualization and development or self-serving [10–12]. Concordantly, prior research has reported that volunteers of self-oriented motivation were fond of volunteering in culture/recreation, environment, law/politics, and business or professional services [11]. Participation in these voluntary services shows that those who volunteer may actually emphasize reciprocation of volunteering to benefit and enhance themselves, e.g. increased social network and ties, understanding of self, evasion of personal problems, acquisition of new skills, and career development [2, 11, 12]. The scores of this form of volunteering also range from 0 to 6, higher scores indicative of more participation in self-oriented volunteering.

### Control variables

The socio-demographic variables adjusted in this study include gender (1 = female, 0 = male), age in years, race/ethnicity, education (1 = none, 2 = high school, 3 = GED, 4 = associates degree, 5 = bachelor’s degree, 6 = graduate degree, 7 = doctorate), citizenship (1 = US citizen, 0 = other), marital status (1 = currently married, 0 = other), number of children at home, annual family income (1 = $0 to $14,999, 2 = $15,000 to $34,999, 3 = $35,000 to $49,999, 4 = 50,000 to $64,999, 5 = $65,000 to $84,999, and 6 = $85,000 or more), and employment status (1 = employed, 0 = other). Three dummy variables were constructed for race/ethnicity, in which African American (Black), Hispanic/Mexican American, and other races or ethnicities were the contrast groups, and non-Hispanic White was the reference category. Number of children was coded 0 to 4 or more children. As family income has the missing values of 34.9%, Expectation Maximization Imputation (EM) was used to replace the missing values rather than mean substitution, which was applied in previous relevant research [18]. Mean substitution will bias the mean distribution and restrict variance. EM, however, may set off these problems by using a two-step iterative process that involves regression analysis and maximum likelihood procedures to allow all available pertinent variables as predictors for imputing missing data [19]. A dummy variable was created to indicate whether the participant had missing information on income (1 = missing, 0 = other), to preclude confounding.

### Statistical analyses

Due to the multi-correlated nature of the health outcomes, the current study employed multivariate linear regression to analyze the results.^1^ This modeling approach has the advantage of reducing multi-collinearity and problems of Type I errors when there are significant correlations among the outcome variables [22]. Then, all five health outcomes are concurrently regressed on the predictors of volunteering and pertinent socio-demographic covariates. For easy interpretation of the regression results, the predictors of volunteering and socio-demographic covariates were standardized into z-scores, so it is possible to calculate percentages increased in the health outcomes by one unit increase in volunteering (e.g. additional participation in the voluntary services) through likelihood ratio= *e* ^*β*^ − 1. In addition, the Wald test of parameters equivalence constraint was used to ascertain whether other-oriented volunteering had stronger health effects than did self-oriented volunteering. The statistical analyses were performed by Mplus 7.11.

## Results

Table 1 presents the correlations of the five health outcomes and the predictors of other-oriented and self-oriented volunteering. Mental and physical health, life satisfaction and social well-being were significantly and positively correlated with each other, rs = .247 to .369, ps < .001, and they were significantly and negatively correlated with depression, rs = −.334 to −.491, ps < .01. The predictors of other-oriented and self-oriented volunteering were significantly correlated with the five health outcomes.

**Table 1:** Correlations of the study variables

		1	2	3	4	5	6	7
1.	Mental health	--						
2.	Physical health	.369**						
3.	Life satisfaction	.425**	.299**					
4.	Social well-being	.142**	.158**	.136**				
5.	Depression	−.463**	−.334**	−.491**	−.358**			
6.	Other-oriented volunteering	.142**	.158**	.136**	221**	−.098**		
7.	Self-oriented volunteering	.132**	.155**	.098**	160**	−.088**	.591**	--

^+^ *p* < .1

*p < .05

**p < .01

Table 2 presents the results of multivariate linear regression of other-oriented volunteering on the five health outcomes. Results showed that other-oriented volunteering was significantly predictive of better mental health, β = .082, physical health, β = .087, life satisfaction, β = .071, and social well-being, β = .106, ps < .01, as well as fewer depressive symptoms, β = −.044, *p* < .05. The results were held even accounting for multiple pertinent socio-demographic variables. Likelihood ratio estimates found that additional participation in voluntary services in the form of other-oriented volunteering resulted in an 8.54% increase in mental health, 9.08% in physical health, 7.35% in life satisfaction, and 11.11% in social well-being, as well as 4.30% decrease in depression, giving evidence that higher participation in voluntary services pertinent to other-oriented volunteering contributes to better health benefits cumulatively.

**Table 2:** Multivariate linear regression of other-oriented volunteering on the health outcomes of mental and physical health, life satisfaction, social well-being, and depression

Predictors		Mental health		Physical health		Life satisfaction		Social well-being		Depression	
		β	t	β	t	β	t	β	t	β	t
1.	Other-oriented volunteering	.082**	3.043	.087**	3.046	.071**	4.225	.106**	5.057	−.044*	−2.472
2.	Female	−.043	−1.668	−.003	−.123	−.003	−.166	.042*	2.088	.023	1.340
3.	Age	−.064*	−2.181	−.188**	−6.068	.059**	3.223	−.052*	−2.281	−.076**	−3.912
	Race/Ethnicity										
4.	Black	−.024	−.929	−.042	−1.538	−.030^+^	−.1839	−.041*	−2.038	.023	1.326
5.	Hispanic	−.092	−3.132	−.116**	−3.708	−.036*	−1.975	−.081**	−3.525	.021	1.100
6.	Other	−.016	−.621	−.048	−1.786	−.004	−.223	.001	.053	.020	1.178
7.	Education	.139**	4.959	.119**	4.003	.059**	3.361	.105**	4.781	−.096**	−5.150
8.	US citizen	−.011	−.379	−.040	−1.353	−.024	−1.334	.079**	3.564	.064**	3.437
9.	Married	.105**	3.891	.069*	2.397	.127**	7.481	.210**	9.854	−.105**	−5.853
10.	Number of children	−.041	−1.439	−.032	−1.084	−.030^+^	−1.688	.005	.231	.004	.211
11.	Employed	.071**	2.620	.110**	3.799	−.005	−.292	.012	.541	−.080**	−4.411
12.	Family income	−.012	−.457	.104**	3.684	.028^+^	1.678	.071**	3.378	−.106	−.922
13.	Income missing	.005	.177	−.048^+^	−1.754	−.021	−1.298	−.051*	−2.491	−.020	−1.174
	R^2^	.076		.119		.106		.196		.103	
	Model chi-square (df)	2164.205(75)**									

^+^ *p* < .1

*p < .05

**p < .01

Moreover, self-oriented volunteering was significantly predictive of better mental health, β = .063, physical health, β = .069, life satisfaction, β = .036, and social well-being, β = .053, ps < .05, but did not significantly predict depressive symptoms, β = −.021, *p* > .05 (Table 3). Likelihood ratio estimates showed that additional participation in voluntary services in the form of self-oriented volunteering resulted in a 6.50% increase in mental health, 7.14% in physical health, 3.66% increase in life satisfaction, and 5.44% increase in social well-being, also giving evidence to the cumulative effects of self-oriented volunteering on health benefits.

**Table 3:** Multivariate linear regression of self-oriented volunteering on the health outcomes of mental and physical health, life satisfaction, social well-being, and depression

Predictors		Mental health		Physical health		Life satisfaction		Social well-being		Depression	
		β	t	β	t	β	t	β	t	β	t
1.	Self-oriented volunteering	.063*	2.364	.069*	2.456	.036*	2.164	.053*	2.547	−.021	−1.219
2.	Female	−.035	−1.376	.005	.172	.003	.204	.051*	2.519	.019	1.125
3.	Age	−.069*	−2.353	−.193**	−6.243	.053**	2.907	−.061**	−2.653	−.072**	−3.732
	Race/Ethnicity										
4.	Black	−.021	−.830	−.039	−1.438	−.028^+^	−1.700	−.038^+^	−1.869	.021	1.248
5.	Hispanic	−.095**	−3.218	−.118**	−3.794	−.039*	−2.095	−.085**	−3.657	.023	1.174
6.	Other	−.015	−.581	−.046^+^	−1.739	−.004	−.224	.001	.505	.020	1.178
7.	Education	.147**	5.318	.127**	4.333	.070**	4.038	.122**	5.594	−.103**	−5.598
8.	US citizen	−.005	−.182	−.035	−1.168	−.017	−.967	.088**	4.002	.060**	3.228
9.	Married	.105**	3.873	.069*	2.381	.127**	7.437	.209**	9.779	−.105**	5.838
10.	Number of children	−.036	−1.274	.027	−.920	−.026	−1.445	.011	.516	.001	.071
11.	Employed	.068*	2.487	.106**	3.654	−.006	.363	.010	.453	−.079**	4.351
12.	Family income	−.016	−.592	.100**	3.522	.027	1.573	.069**	3.239	−.015	−.868
13.	Income missing	.007	.283	−.045	−1.639	−.019	−1.194	−.048*	−2.359	−.021	−1.226
	R^2^	.074		.117		.099		.185		.101	
	Model chi-square (df)	2140.092(75)**									

^+^ *p* < .1

*p < .05

**p < .01

The Wald test of parameters equivalence constraint was performed to examine whether the effects of other-oriented and self-oriented volunteering on health outcomes were significantly different. The predictors of other-oriented and self-oriented volunteering were first put into two regression equations separately while retaining all the socio-demographic covariates in the multivariate linear regression models intact. Then, the two regression equations were simultaneously pooled in a single comparison model and the parameters of other-oriented and self-oriented volunteering were set to be equivalent (β_other-oriented_ = β_self-oriented_). Results showed that other-oriented volunteering had significantly stronger effects on the health outcomes of mental and physical health, life satisfaction, and social well-being than did self-oriented volunteering (Table 4), but they did not differ in the effect on depression. The strongest different effect was for social well-being (difference in betas), then life satisfaction, and mental and physical health. The beta differences range from .053 to.018.

**Table 4:** Results of parameters equivalence constraints for effects of other-oriented and self-oriented volunteering on health outcomes^a^

	Health Outcomes	Other-oriented volunteering	Self-oriented volunteering	Difference in betas^b^	Wald X^2^
		β (SE)	β (SE)		
1	Mental health	.082 (.027)	.063 (.027)	.019	9.237**
2	Physical health	.087 (.028)	.069 (.028)	.018	13.797***
3	Life satisfaction	.071 (.017)	.036 (.017)	.035	4.139*
4	Social well-being	.106 (.021)	.053 (.021)	.053	9.735***
5	Depression	−.044 (.018)	−.021 (.018)	.023	.029

^a^The socio-demographic variables adjusted in the multivariate linear regression models were also controlled in the parameters equivalence constraints model

^b^Difference in Betas is simply (β_other-oriented_ – _βself-oriented_); β = regression beta; SE = stand error

*p < .05

**p < .01

***p < .001

## Discussion

The present study investigated the cumulative effects of other- and self-oriented volunteering on various health outcomes in a population-based sample of general adults, a previously uncharted research topic. Results confirmed that volunteering, regardless of the form being examined, had significant health effects. Past pertinent research investigating the volunteering and health connection mainly focused on a subpopulation of people, e.g. elderly people, or disregarded the interrelated relationships between the health outcomes, hence compromising external validity and accuracy of the results [7–9]. Results of this study add evidence to the literature that volunteering engenders health benefits across various health outcomes in a cumulative way by participation in several voluntary services contemporaneously in the general adult public. Consistent with prior research, the present study supports the beneficial effects of volunteering on mental and physical health, life satisfaction, social well-being and depression. However, despite the cumulative health effects of volunteering across various health outcomes found in this study, the magnitude of these health effects did vary. Most notable are the strongest effect of other-oriented volunteering on social well-being and the strongest effect of self-oriented volunteering on physical health, which reveal the different nature of these two forms of volunteering. Other-oriented volunteering is more other-regarding, altruistic and humanitarian-concerned than is self-oriented volunteering; the latter is more self-enhancing and self-actualizing [10, 11]. Thus, more trustworthy interpersonal relationships, a supportive network, a sense of mattering and life meaning are expected for other-oriented volunteering rather than self-oriented volunteering. In contrast, self-oriented volunteering may involve more physical, cultural and career activities that may maximize the physical health of volunteers. However, these postulates do not negate the also robust positive effect of other-oriented volunteering on physical health, and the above explanations are tentative. Hence, more research is needed.

In addition, the Wald test of the parameters equivalence constraint supports the stronger health effects of other-oriented volunteering rather than of self-oriented volunteering, indicating that serving others out of sheer altruism, genuineness and humanitarian concern is important in reaping better health. The serving process of other-oriented volunteering stresses unselfishness, sharing, other-directedness, and generosity, which are counteractive to the ego-centric and self-serving culture that is upheld nowadays and may harm mental and behavioural health [23]. In fact, some personal intrinsic motives, e.g. narcissism and self-preoccupation, may lead to health-compromising behaviour and then detriment to health [23, 24]. Recent mental health research supports the importance of some virtues, e.g. generosity and gratitude, in relation to health [25, 26]. Therefore, it is plausible that the health effects of other-oriented volunteering are significantly stronger than those of self-oriented volunteering found in this study (Table 4). In addition, this difference in intrinsic motives between other-oriented and self-oriented volunteering helps explain why the former can alleviate depression but the latter does not, as self-preoccupation and pursuits are etiologic of depression [24].

However, when comparing the effects of other-oriented and self-oriented volunteering on depression, the Wald test did not find a significant difference between the two forms of volunteering. This corresponds to the eudaimonic theory of well-being and past research results of volunteering effects on positive and negative affect [8, 27]. These results indicate that engaging in meaningful and prosocial behaviours, e.g. volunteering, may effectively enhance positive emotions but may be less efficacious in reducing negative affect or mental distress. This may be due to volunteering that, regardless of the form, is not a direct problem-solving strategy to tackle and resolve negative affect, that is, caused by a specific life situation such as traumatic events and experiences. Hence, the weakest significant effect of other-oriented volunteering, β = −.044, *p* < .05, and the weakest and insignificant effect of self-oriented volunteering on depression, β = −.021, *p* > .05, are evident in this study. Thus, it is comprehensible to have an insignificant difference when comparing the effects of the two forms of volunteering on depression. Nevertheless, future research should put a lens on different health effects of volunteering on the positive and negative side of health outcomes.

Recent research studies have reported that other-oriented volunteers tend to be more involved, satisfied and persistent in their volunteering work than are self-oriented volunteers [11, 28]. Therefore, when promoting the health effects of volunteerism, health professionals, educators and policymakers should note the importance of volunteers of different orientations in influencing the sustainability and provision of services. In fact, a better matching strategy is needed for the alignment of appropriate types of voluntary services to volunteers of different orientations [28, 29]. As the results of better health benefits by other-oriented volunteering obtained from this investigation and other pertinent studies show [2, 11, 29], related parties and organizations should highlight the health benefits of serving others in need with altruistic attitudes and humanitarian concerns and promote an other-regarding culture of volunteerism. Table 1 shows a significant correlation between other-oriented and self-oriented volunteering, implying that some volunteers may cut across and simultaneously participate in other- and self-oriented forms of voluntary services. Hence, future research should explore if this mixed form of volunteering might result in comparatively better health benefits than other forms might.

In this study, the adult participants who were older, non-White, had less education, were unmarried, and unemployed had poorer health outcomes across both the other-oriented and the self-oriented volunteering regression models. In fact, people with these background characteristics also tend to volunteer less [3, 7, 14], which would occasion a twofold effect on their health risks. Hence, promoting volunteering opportunities to these people can be a way of keeping them healthy.

## Conclusion

Public health, education and policy practitioners are advised to encourage volunteering as a kind of healthy lifestyle among the general public, especially in the form of other-oriented volunteering. They should have social service professionals promote a culture of volunteerism among underprivileged social groups, e.g. elderly people, ethnic minorities, lower-educated people, unmarried and unemployed people. Although there has been a changing trend toward episodic and self-oriented volunteering in recent years [11, 27, 28], highlighting the better health effects of other-oriented volunteering and promoting the basic altruistic and other-regarding nature of volunteerism should be noted for the related practitioners. However, the present study has certain limitations. First, cross-sectional data make causality of the relationships impossible. Second, self-reported health outcomes are less favourable than are the objectively diagnosed health outcomes. Third, broad classification of participation in various types of voluntary services into other- and self-oriented volunteering based on secondary data is less adequate than are first-hand data, which can more effectively help clarify the nature of voluntary services for classification purposes. Hence, it is necessary to be aware of the limitations of the classification approach based on the secondary data used in this study. Lastly, neither the present investigation nor most prior studies have explored possible mediators that link the relationship between volunteering and health, which is important for comprehension of the mechanisms that volunteering engenders on health benefits. Therefore, future studies should address these limitations and provide a more comprehensive picture of the health benefits of volunteering.

## Abbreviations

EM: Expectation Maximization
GED: General Educational Development
US citizen: citizen of the United States

## Footnotes

1 Three of the five health outcome variables were measured by a single question item with a 4-point or a 5-point scale, and some researchers believe this type of outcome should be treated as ordered categorical variables. Therefore, we re-ran the data with Probit regression models. Results showed that the standardized coefficients of Probit regression models were similar to the findings obtained from multivariate linear regression models. In fact, statisticians have argued that when a dependent variable is measured by 4-point or 5-point rank-ordered categories, there would be little difference between the results obtained from ordinal regression and OLS regression [20, 21]. When an outcome is measured by an ordered categorical scale, e.g. “Overall, how would you rate your mental health at the present time? Would you say it is excellent, very good, good, fair, or poor?” on a 5-point scale (1 = excellent, 2 = very good, 3 = good, 4 = fair, and 5 = poor), a latent continuous variable, y^*^, ranging from -∞ to ∞ is denoted. For measurement purposes this is, mapped to an observed variable y. Hence, the above ordinal outcome as an example is in principle related to a continuous, latent variable y^*^ in indicating a person’s level of mental health by category, ranging from “excellent” to “poor”. Therefore, the observed y is related to y^*^ in an equation of “*y*_*i*_ *= m if τ*_*m-1*_ *≤ y*_*i*_^***^ *<τ*_*m*_ *for m = 1 to Ј*”. For easier interpretation, the present study opted for the results of multivariate linear regression models. Readers can contact the first author to obtain the results from Probit regression models for reference.
